# Long-Term Anabolic Androgenic Steroid Use Is Associated with Increased Atrial Electromechanical Delay in Male Bodybuilders

**DOI:** 10.1155/2014/451520

**Published:** 2014-05-04

**Authors:** Mustafa Akçakoyun, Elnur Alizade, Recep Gündoğdu, Mustafa Bulut, Mehmet Mustafa Tabakcı, Göksel Açar, Anıl Avcı, Zeki Şimşek, Serdar Fidan, Serdar Demir, Ramazan Kargın, Mehmet Yunus Emiroğlu

**Affiliations:** ^1^Cardiology Department, Kartal Kosuyolu Heart Research and Training Hospital, Denizer Street, Kartal, 34846 Istanbul, Turkey; ^2^Cardiology Department, Fatih University, Yalı Street, Maltepe, 34844 Istanbul, Turkey; ^3^Cardiology Department, Kartal Yavuz Selim State Hospital, Yukarı Street, Kartal, 34860 Istanbul, Turkey

## Abstract

We investigated the effect of long-term supraphysiologic doses of anabolic androgenic steroids (AAS) on atrial electromechanical delay (AEMD) in male bodybuilders. We clearly demonstrated that long-term consumption of supraphysiologic doses of AAS is associated with higher values of inter- and intra-AEMD in healthy young bodybuilders.

## 1. Introduction 

Self-administration of high doses of anabolic androgenic steroids (AAS) is a widespread practice among athletes to increase lean body mass and muscular strength. Long-term illicit use of supraphysiologic doses of AAS may cause several adverse cardiovascular effects [1–4]. Recent studies have found pathological left ventricular (LV) hypertrophy, diastolic dysfunction, and subclinical LV systolic impairment in long-term AAS users [4–7]. In addition, ventricular and atrial arrhythmic events were described secondary to the intake of AAS. Atrial fibrillation (AF) is the most frequently observed arrhythmia in bodybuilders who are using AAS [8]. Moreover, various case reports of AF among AAS users suggest a causal link between AAS use and AF in power athletes [8–11]. However, the mechanisms underlying such predispositions to AF are poorly understood and also it is not clear that AAS using athletes are more prone to atrial rhythm disturbances than non-AAS users.

The prolongation of intra-atrial and interatrial conduction times and the inhomogeneous propagation of sinus impulses are typical electrophysiological features of the atrium which is prone to fibrillate [12, 13]. Moreover, atrial electromechanical delay (AEMD) as measured by tissue Doppler imaging (TDI) has been shown to detect atrial impairment in paroxysmal AF [13, 14]. Another important point is that AEMD may also predict the development of new-onset AF [15]. Since AF is a reentrant arrhythmia, it is logical that the triggering factor generally is a critically timed atrial activation that may give rise to reentry in a vulnerable structure [16]. Atrial and ventricular structural alteration, increased atrial stretch, autonomic imbalance, atrial interstitial fibrosis, inflammation, and ischemia may act in this respect as an internal or external factor by modulating atrial refractoriness through both atria and modifying intra-atrial conduction [17–19]. Because these factors are effected by long-term use of supraphysiologic doses of AAS [10, 20, 21], there might be an association between AAS use and AEMD. Changes in inter-AEMD and intra-AEMD in the AAS using athletes have not been investigated previously. Therefore, we attempted to investigate atrial conduction abnormalities in AAS using athletes and to compare those of non-AAS users by using electromechanical coupling interval and TDI.

## 2. Methods

### 2.1. Study Population

We selected a population of 33 competitive bodybuilders, including 15 who actively used AAS for ≥2 years (users) and 18 who had never used AAS (nonusers), all men. Written informed consent was obtained from each subject, and the study was approved by the appropriate institutional ethics review committee. Exclusion criteria were presence of coronary artery disease, valvular or congenital heart disease, hypertension, congestive heart failure, diabetes mellitus, sinus tachycardia, psychiatric, respiratory, or metabolic disorders, inadequate echocardiographic quality, and smoking habit.

#### 2.1.1. Training Protocols

All participants had trained intensively for >10–15 h/wk for >5 years. AAS users and nonusers had started bodybuilding at approximately the same age (21.61 ± 3.04 versus 22.34 ± 3.68 years, resp., *P* = NS) and completed the same anaerobic isometric static exercises (4.94 ± 1.82 versus 4.73 ± 2.02 h/wk, *P* = NS). Maximum self-reported one-repetition squat results were significantly greater among AAS users (142.67 ± 19.09 versus 120.67 ± 21.61 kg, *P* < 0.05; Table 1).

#### 2.1.2. AAS Abuse

An anonymous, self-administered questionnaire was used to investigate each athlete's clinical (diseases and medication) and drug intake history (type and timing of steroid use and other performance-enhancing drugs). Additionally, urine testing was performed by high-performance liquid chromatography coupled to mass spectrometry to confirm or exclude any recent consumption of anabolic steroids. Each AAS user admitted the current use of multiple AAS administered by intramuscular injection and/or orally. The orally self-administered drugs were oxymetholone and stanozolol, and the injectable steroids were nandrolone, stanozolol, and testosterone propionate. The mean duration of AAS use was 5.73 ± 3 years (range, 4–20 years). The mean weekly dosage of AAS was 1085.5 ± 354 mg.

#### 2.1.3. Physical Examination and Laboratory Tests

All subjects were examined on an empty stomach. Height, weight, body mass index (BMI) (kg/m^2^), body surface area (BSA) (m^2^), heart rate, and blood pressure were measured. Venous blood samples were drawn from each subject, always in the afternoon between 1 and 2 PM, to evaluate serum hormone levels (testosterone, luteinizing hormone, follicle-stimulating hormone, insulin, T3, and T4), hematology (hematocrit, hemoglobin), and blood lipids (total cholesterol, high-density lipoprotein).

### 2.2. Echocardiographic Measurements

Echocardiography was performed in left lateral decubitus position with an ultrasound machine GE-Vingmed Vivid 7 system (Vivid system 7, GE-Vingmed Ultrasound AS, Horten, Norway) and 3S-RS (3.5 MHz) probe. Examinations were performed by a cardiologist who was blinded to the clinical details of each subject. Single-lead ECG was recorded continuously during the echocardiographic examination. Two-dimensional, M-mode and tissue Doppler images were acquired from the parasternal long and short axis and apical four-chamber views at end-expiratory apnea and were transferred to customized dedicated software package (EchoPAC, General Electric Vingmed Ultrasound) for offline analysis of stored data. All measurements were averaged from three cardiac cycles. 2D echocardiographic measurements were performed according to standards outlined by the American Society of Echocardiography [22]. Left atrium (LA), LV dimensions, and wall thickness were obtained from the parasternal long axis with an M-mode cursor positioned just beyond the mitral leaflet tips, perpendicular to the long axis of the ventricle. LV end-diastolic diameter (LVEDD) and end-systolic (LVESD) diameter, thickness of the interventricular septum (IVS), and posterior wall of the left ventricle (PW) were measured. LV ejection fraction was calculated according to the Simpson method [22]. For determination of LVM, the Devereux formula was used: LVM (g), 0.8 (1.04 ([LVIDD + PWTD + IVSTD]^3^ − [LVIDD]^3^)) + 0,6 (LVID indicates LV internal dimension; PWT, PW thickness; IVST, IVS thickness) [23]. Left ventricular mass index was calculated by dividing LVM by body surface area. LV hypertrophy was defined as an LV mass index >115 g/m^2^ in men, as recommended by the ASE and the EAE. The calculation of relative wall thickness (RWT) was performed using the formula (2 × PW)/LV internal dimension [22]. LA areas and volumes were measured in the apical four-chamber and two-chamber views at ventricular end-systole (maximum LA size) and mean values of area and volume were obtained. LA mean volume was indexed to body surface area (BSA) [22]. Mitral inflow velocities were evaluated by pulsed-wave Doppler echocardiography with the sample volume placed at the tip of the mitral leaflets from the apical four-chamber view. Diastolic peak early (*E*) and peak late (*A*) transmittal flow velocity, peak *E* to peak *A* velocities (*E*/*A*), and isovolumic relaxation time (IVRT) were measured [24].

TDI was performed in the apical four-chamber view using a 5 mm pulsed Doppler sample volume with as minimum optimal gain as possible to obtain the best signal-to-noise ratio. Care was taken to align the echo image so that the annular motion was parallel to the TDI cursor. Spectral pulsed-wave Doppler signal filters were adjusted until a Nyquist limit of 15–20 cm/s was reached. The monitor sweep speed was set at 50–100 mm/s to optimize the spectral display of myocardial velocities. In apical 4-chamber view, the pulsed Doppler sample volume was subsequently placed at the level of LV lateral mitral annulus, septal mitral annulus, and right ventricular (RV) tricuspid annulus. The myocardial peak systolic (*S*_*m*_) and early diastolic (*E*_*m*_) velocity and late diastolic (*A*_*m*_) velocity were obtained from the septum, the lateral wall of the left ventricle, and the annulus of the right ventricle. The Em global and Am global velocities were derived by averaging the velocities from the 2 mitral annular sites. Global *E*_*m*_/*A*_*m*_ ratio and *E*/*E*_*m*_ ratio were calculated [25].

### 2.3. Atrial Electromechanical Delay

**Figure 1:**
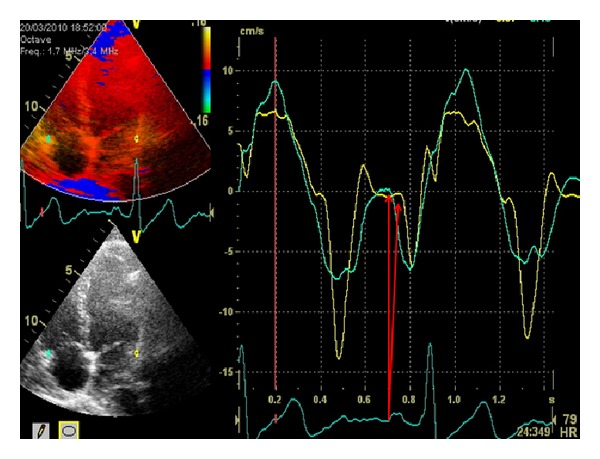
Pulsed-wave tissue Doppler early diastolic [*E*_*m*_] and late diastolic [*A*_*m*_] mitral and tricuspid annular velocities. The measurement of PA is determined by the onset of the P wave to the onset of *A*_*m*_ and illustrated with the arrows.

Atrial electromechanical delay (AEMD) was measured as the interval between the onset of the P wave on the electrocardiogram and the beginning of late diastolic Am wave at the lateral mitral annulus (PA (atrial electromechanical coupling) lateral), septal mitral annulus (PA septum), and RV tricuspid annulus (PA tricuspid). Values were averaged over three consecutive beats. The difference between PA lateral and PA tricuspid (PA lateral, PA tricuspid) was defined as inter- AEMD and the difference between PA septum and PA lateral (PA septum, PA lateral) was defined as intra-AEMD [26] (Figure 1). In AEMD measurements, intraobserver variability was assessed in 20 selected subjects at random from the patient study group by repeating the measurements under the same basal conditions. To test the interobserver variability, we performed the measurements offline from video recordings by a second observer. The intraobserver and interobserver variability for TDI calculated from 20 consecutive patients were 5.7% and 4.7% for PA lateral, 5.6% and 4.8% for PA septum, and 5.7% and 5.3% for PA tricuspid, respectively.

### 2.4. Statistical Analysis

Continuous variables are expressed as mean ± standard deviation and categorical data are expressed as percentages. Statistical comparison of quantitative data was performed by unpaired *t*-test. The correlation analyses between continuous variables were performed by Pearson's correlation analysis. Multiple regression analysis was used to identify significant predictors of inter- and intra-AMED. Thus, all predetermined independent variables that correlated with a *P* value of less than <0.05 in the Pearson correlation analysis were inserted into a stepwise, multiple regression analysis. A *P* value of <0.05 was considered statistically significant. All statistical studies were carried out with the SPSS program (version 16.0, SPSS Inc., Chicago, IL, USA).

## 3. Results

### 3.1. Clinical Characteristics of the Study Population

The characteristics of the subjects are listed in Table 2. No differences between groups emerged from age, height, weight, BSA, blood pressure, or heart rate. However, AAS users had higher body mass indexes compared with AAS nonusers.

### 3.2. Echocardiographic Analysis

Table 3 shows the details of the echocardiographic analysis. LV mass index, interventricular septal thickness, LV posterior wall thickness, and relative diastolic wall thickness were significantly greater in AAS users than in nonusers and sedentary controls (*P* < 0.01). No significant differences were found in LA, LA volume index, LV end-systolic, end-diastolic dimensions, and LV ejection fraction among the groups.

Transmitral Doppler echocardiography data of LV diastolic function are listed in Table 2. No significant differences were found in peak *E* and peak *A* between AAS users and nonusers. However, drug-using bodybuilders exhibited longer isovolumetric relaxation times and lower ratio of *E*/*A* than their drug-free counterparts.

When comparing the diastolic functions obtained by measuring the TDI velocities, lateral and septal *E*_*m*_ were significantly lower in AAS users than in nonusers (11.6 ± 1.2 versus 16.2 ± 1.5,  *P* < 0.01; 10.1 ± 1.3 versus 12.1 ± 1.5, *P* < 0.01; resp.), whereas lateral and septal *A*_*m*_ were not a significant difference in AAS users compared to nonusers (9.4 ± 1.3 versus 9.9 ± 1.2, *P* > 0.05; 9.5 ± 0.7 versus 9.4 ± 1.2, *P* > 0.05, resp.). Global *E*/*E*_*m*_ and *E*_*m*_/*A*_*m*_ were significant difference in ASS users compared to nonusers (7.3 ± 1.5 versus 5.8 ± 0.9, *P* < 0.01; 1.6 ± 0.1 versus 1.5 ± 0.2, *P* < 0.01, resp.).

### 3.3. Atrial Electromechanical Delay Parameters

Table 4 shows the atrial electromechanical intervals measured at the lateral, septal, and RV annulus by the tissue Doppler method. The PA lateral and PA septum were significantly higher in the AAS user than in nonusers (65.55 ± 7.50 versus 49.08 ± 6.66, *P* < 0.01; 49.27 ± 7.88 versus 42.71 ± 4.39, *P* < 0.01, resp.). Interatrial and intra-atrial EMD values were significantly higher in the AAS using bodybuilders compared with those in the nonusers (26.15 ± 6.54 versus 12.42 ± 6.58, *P* < 0.01; 9.88 ± 5.23 versus 6.04 ± 3.21, *P* < 0.05, resp.). There was a positive correlation between LV mass index and inter-AMED (*r* = 0.430, *P* < 0.012) and intra-AMED (*r* = 0.381, *P* < 0.029). There was also correlation between global *E*/*E*_*m*_, global *E*_*m*_/*A*_*m*_, and inter-AMED (*r* = 0.436, *P* < 0.011; *r* = −0.406, *P* < 0.019, resp., Table 5). The linear regression analysis revealed that AAS using was an independent predictor of only inter-AMED (*P* < 0.001).

## 4. Discussion

In this study, we used a novel noninvasive technique to show inter- and intra-AEMD by TDI. In our study, we found that inter-AEMD and intra-AEMD are significantly increased in AAS using bodybuilders compared with nonusers that are known to be related to various arrhythmias, especially AF. This is the first study evaluating inter- and intra-AEMD in AAS user and nonuser bodybuilder athletes.

Previous studies reported that long-term illicit use of supraphysiologic doses of AAS was associated with reduced LV diastolic functions (impaired relaxation and reduced compliance of LV), increased LV mass, LV/atrial hypertrophy, subclinical systolic impairment, increased myocardial stiffness and myocardial fibrosis, and altered cardiac autonomic system regulation [4–7, 20, 21, 27]. Furthermore, it has been reported that myocardial infarction, cardiomyopathy, sudden death, cardiovascular morbidity, and mortality have significantly increased in long-term AAS using bodybuilders more than nonusers [28]. In addition, arrhythmic events were described secondary to the long-term intake of AAS. Although AF is the most frequently observed arrhythmia, ventricular arrhythmias were also described [8–11, 29]. However, it is not clear that AAS using bodybuilders are more prone to rhythm disturbances compared with nonusers.

The prolongation of intra- and inter-AEMD and the inhomogeneous propagation of sinus impulses are well-known electrophysiologic characteristics of the atria which is prone to fibrillation [12, 13]. The evaluation of AEMD by using TDI has been studied in patients with rheumatic mitral stenosis, paroxysmal AF, acute sleep deprivation, and type I diabetes mellitus [26, 30–32]. Also, Roshanali and colleagues have found that atrial electromechanical interval is a predictor of AF emerging after coronary artery bypass grafting and demonstrated that the preoperative administration of amiodarone to patients having longer atrial electromechanical interval has decreased the postoperative atrial fibrillation incidence [33]. Furthermore, De Vos et al. showed that prolonged PA-TDI interval (indicator of AEMD) predicted the development of new-onset AF in their study, which included 249 patients [15]. In addition, prolonged AEMD in patients with paroxysmal AF was reported with TDI and pulsed-wave Doppler echocardiographic studies [14, 30]. In the present study, we found that inter- and intra-atrial AEMD were prolonged in AAS users compared with both nonusers.

There may be several mechanisms involved in increasing inter-AEMDs in chronic consumption of supraphysiologic doses of AAS. There are several studies that indicate impairment of LV diastolic function, which is known to play a role in the pathogenesis of AF [17, 34, 35], which was also found to be impaired in AAS using athletes [4–7]. When left ventricular diastolic dysfunction occurs, emptying of the left atrium is impaired as well. Following impaired left ventricular diastolic relaxation, there is increased atrial contribution to the mitral flow in the left ventricular diastolic flow, thus leading to atrial overstretching and enlargement [36]. The left atrium diameter is known to be correlated with cardiovascular events and is a risk factor for AF [37]. In this study, the left atrial diameters of the AAS user and nonuser groups were similar. However, the presence of left ventricular diastolic dysfunction in AAS user athletes is a controversial issue. Pearson et al. and De Piccoli et al. described an impaired diastolic function in weightlifters taking AAS when compared to AAS-free counterparts [38, 39]. Other authors did not find any difference in the diastolic function of strength athletes with or without AS abuse [40–42]. The studies mentioned above used two-dimensional echocardiography and Doppler measurements of transmitral blood flow to assess diastolic function. In our study this technique was not able to show an altered diastolic function in AAS users too. The discrepancies between these studies could be attributed to the duration and/or the intensity of the training programs, among the groups of AAS users included in these studies and AAS dosage [38, 42, 43]. We investigated the diastolic functions by using the tissue TDI method as well because the conventional Doppler method is load dependent and TDI constitutes a good index of LV relaxation properties. In previous studies, the *E*/*E*_*m*_ and *E*_*m*_/*A*_*m*_ were demonstrated to be significantly correlated with the left ventricle end-diastolic pressure and diastolic dysfunctions [24, 44]. In our study, we found that *E*/*E*_*m*_ ratio was significantly higher in AAS users than in nonusers. In addition, the *E*_*m*_/*A*_*m*_ ratio was significantly lower in AAS users than in nonusers. Also we found that IVRT prolonged in AAS using group, indicating the impartment of diastolic function. Furthermore, we found correlations between *E*/*E*_*m*_ and *E*_*m*_/*A*_*m*_ and inter-AMED [*r* = 0.436; *P* = 0.011 and *r* = −0.406; *P* = 0.019, resp.] in our study. Therefore, we believe that this impairment in the diastolic function might be one of the reasons for the prolonged atrial conduction times in AAS using bodybuilders.

The other possible mechanism for increasing inter-AEMD and intra-AEMD in AAS using athletes is LV pathological hypertrophy. LV pathological hypertrophy induced by AAS appears to be generated by a direct action on cardiac androgen receptors, whose effects are directly proportional to the doses, time, and duration of drug administration [4]. In our study LV wall thickness and LV mass index were enlarged in AAS using athletes compared to nonusers. The presence of LV hypertrophy is an indicator of increased myocardial demand for oxygen and hence decreases coronary reserve. When coronary blood flow is fixed or reduced, there is a supply-demand mismatch, resulting in increased risk for ischemia. In such a scenario, a decrease in blood flow can be catastrophic to the already increased demand of the myocardial cells. Patients with LV pathological hypertrophy are at increased risk for ischemia, probably causing prolongation of inter- and intra-AEMD [34]. Yavuz et al. showed positive correlation between LV hypertrophy and atrial conduction delay in hypertensive patients [34]. Similarly, we found a positive correlation between LV mass index and inter- and intra-AMED in our study AMED [*r* = 0.430; *P* = 0.012 and *r* = 0.381; *P* = 0.029, resp.].

Probably adverse effects of AAS on the cardiovascular system are also due to direct toxicity on myocardial structure with increased collagen deposition, fibrosis, and altered microcirculation with intimal hyperplasia of the intramural coronary arteries resulting in chronic ischemic damage [21]. Vascular endothelial cells may be directly affected by AAS, which may result in vasospasm [21]. As the cause of these alterations, AAS may directly affect the atrium, causing heterogeneity in the atrial conduction [20, 21]. In our study, after the linear regression analysis, AAS using in bodybuilders was the independent predictor of the inter-AMED. Therefore, we speculated that long-term illicit use of supraphysiologic doses of AAS might directly affect atrial conduction time (inter-AMED). The last possible mechanism to increase AEMD may be sympathetic activation. It has been shown that chronic consumption of supraphysiologic doses of AAS induces cardiac autonomic imbalance by reduction in parasympathetic cardiac modulation and increase in sympathetic cardiac modulation [10]. Experimental studies showed that greater sympathetic activation leads to myocardial injury. Increased sympathetic activity may also trigger atrial arrhythmias [45]. Therefore, altered autonomic system regulation occurring secondary to the chronic consumption of supraphysiologic doses of AAS may be the other reason underlying the delayed interatrial electromechanical coupling intervals.

## 5. Study Limitations

Our study has several limitations. The most important limitations of our study are the small sample size and cross-sectional design of the study, in which we could not follow up the patients prospectively for future arrhythmic events. We did not observe any arrhythmias in the study population. Therefore, we do not know whether atrial electromechanical delay predicts atrial arrhythmias in chronic consumption of supraphysiologic doses of AAS user athletes. Further studies need to be conducted with a larger number of patients and a longer follow-up time in order to increase the accuracy of the results.

We were dealing with young individuals. Thus, the impact of AAS on AMED in older individuals is unknown. The same idea can be used for gender. There is no guarantee that the effects of AAS on atrial electromechanical delay in women are similar to those found in men. The information about the intake of steroids was self-reported, but it is difficult to assess this in an objective manner. It seems unlikely that the small differences in AAS intake could explain our results. Finally, training-related influences are also improbable as an explanation for the differences between the AAS users and nonusers in our study, as the training protocol was the same for all the athletes.

## 6. Conculusion

In conclusion, in this cross-sectional study, we clearly found that long-term consumption of supraphysiologic doses of AAS is associated with higher values of inter- and intra-AEMD in healthy young bodybuilders, which suggest that there might be a link between AAS use and atrial fibrillation development and/or recurrence. These findings may be markers of subclinical cardiac involvement in AAS using bodybuilders. Finally, this implication deserves further studies for clarifying the possible linkage between long-term consumption of supraphysiologic doses of AAS bodybuilders and atrial arrhythmias.

## Figures and Tables

**Table 1:** Training programs of the AAS user and nonuser bodybuilders.

	AAS nonusers (*n* = 18)	AAS users (*n* = 15)	*P* value
Sessions per week	3.92 ± 0.86	3.67 ± 0.84	NS
Years	8.64 ± 2.11	9.03 ± 1.94	NS
Starting age	22.34 ± 3.68	21.61 ± 3.04	NS
Anaerobic exercise (h/wk)	4.73 ± 2.02	4.94 ± 1.82	NS
Aerobic exercise (h/wk)	3.11 ± 3.03	1.94 ± 1.82	NS
Maximal weight (kg)	120.67 ± 21.61	142.67 ± 19.09	<0.05

NS: nonsignificant.

**Table 2:** Clinical characteristics of AAS user and nonuser bodybuilders.

Clinical variables	AAS nonusers (*n* = 18)	AAS users (*n* = 15)	*P* value
Age (year)	33.8 ± 4.1	32.5 ± 6.6	NS
Height (cm)	180.4 ± 6.9	179.9 ± 7.3	NS
Weight (kg)	87.4 ± 10.3	90.8 ± 6.3	NS
BMI (kg/m^2^)	26.3 ± 3.2	29.1 ± 4.4	<0.05
BSA (m^2^)	2.08 ± 0,14	2.1 ± 0,14	NS
Blood pressure (mmHg)	120 ± 13.37/80.37 ± 6.49	118.51 ± 9.88/78.51 ± 6.9	NS
Heart rate (beats/min)	68.74 ± 10.45	72.22 ± 13.40	NS

NS: nonsignificant.

**Table 3:** Comparison of the echocardiographic parameters of the subjects: both AAS user and nonuser bodybuilders.

	AAS nonusers (*n* = 18)	AAS users (*n* = 15)	*P* value
2D echocardiographic parameters			
LA dimension (mm)	33.1 ± 0.3	34.2 ± 0.2	NS
LA volume index (mL/m^2^)	26.2 ± 2.3	27.6 ± 2.4	NS
LV end-systolic diameter (mm)	31.9 ± 4.4	33.2 ± 3.2	NS
LV end-diastolic diameter (mm)	49.7 ± 1.9	51.2 ± 3.1	NS
Septal wall thickness (mm)	11.5 ± 1.2	12.4 ± 1.3	<0.01
Posterior wall thickness (mm)	9.8 ± 0.9	11.3 ± 0.7	<0.01
RWT	0.39 ± 0.03	0,44 ± 0,02	<0.01
LV mass index (g/m^2^)	90.9 ± 10.8	113.6 ± 13.6	<0.01
LV ejection fraction (%)	61.37 ± 1.6	60.87 ± 2.3	NS
Doppler parameters			
Peak *E* velocity (m/s)	79.8 ± 9.4	77.6 ± 11.6	NS
Peak *A* velocity (m/s)	55.7 ± 8.9	50.7 ± 6.8	NS
*E*/*A* ratio	1.47 ± 0.3	1.54 ± 0.2	NS
IVRT (ms)	80.7 ± 5.8	83.58 ± 11.7	<0.01
Septal *E*_*m*_ (cm/s)	12.1 ± 1.5	10.1 ± 1.3	<0.01
Septal *A*_*m*_ (cm/s)	9.4 ± 1.2	9.5 ± 0.7	NS
Septal *E*/*E*_*m*_ (cm/s)	6,7 ± 1,2	7,8 ± 1,7	<0,01
Septal *E*_*m*_/*A*_*m*_ (cm/s)	1.29 ± 0.2	1.06 ± 0.2	<0.01
*E*_*m*_ lateral (cm/s)	16.2 ± 1.5	11.6 ± 1.2	<0.01
*A*_*m*_ lateral (cm/s)	9.9 ± 1.2	9.4 ± 1.3	NS
*E*/*E*_*m*_ lateral (cm/s)	4.9 ± 0.8	6.8 ± 1.3	<0.01
*E*_*m*_/*A*_*m*_ lateral (cm/s)	1.6 ± 0.3	1.2 ± 0.2	<0.01
Global *E*/*E*_*m*_ (cm/s)	5.8 ± 0.9	7.3 ± 1.5	<0.01
Global *E*_*m*_/*A*_*m*_ (cm/s)	1.5 ± 0.2	1.6 ± 0.1	<0.01

NS: nonsignificant.

**Table 4:** Comparison of the atrial electromechanical parameters of the subjects: both AAS user and nonuser bodybuilders.

Atrial electromechanical parameters	AAS nonuser (*n* = 18)	AAS user (*n* = 15)	*P* value
PA lateral (ms)	49.08 ± 6.66	65.55 ± 7.50	<0.01
PA septum (ms)	42.71 ± 4.39	49.27 ± 7.88	<0.01
PA tricuspid (ms)	36.66 ± 3.64	39.39 ± 5.75	NS
PA lateral, PA tricuspid* (ms)	12.42 ± 6.58	26.15 ± 6.54	<0.01
PA septum, PA tricuspid** (ms)	6.04 ± 3.21	9.88 ± 5.23	<0.05

NS: nonsignificant.

*Interatrial electromechanical delay. **Intra-atrial electromechanical delay.

**Table 5:** Pearson's correlation analysis (*R* and *P* values) between LV mass index, global *E*/*E*_*m*_, global   *E*_*m*_/*A*_*m*_, and atrial electromechanical parameters.

	Inter-AEMD		Intra-AEMD	
	*R* (coefficient)	*P* value	*R* (coefficient)	*P* value
LV mass index	0.430	0.012	0.381	0.029
Global *E*/*E*_*m*_	0.436	0.011	0.288	0.104
Global *E*_*m*_/*A*_*m*_	−0.406	0.019	−0.194	0.281
